# The effect of caffeine on cerebral asymmetry in rats

**Published:** 2015

**Authors:** M Voiculescu, A Segarceanu, M Negutu, I Ghita, I Fulga, OA Coman

**Affiliations:** * Department of Pharmacology and Pharmacotherapy, Faculty of Medicine, “Carol Davila”University of Medicine and Pharmacy, Bucharest, Romania

**Keywords:** caffeine, EEG, cerebral asymmetry, alpha 1, alpha 2

## Abstract

EEG recordings reflect the gross electrical activity emanating from synaptic currents of individual neurons across large cortical areas. During periods of cortical activation, waking, and higher EEG frequencies, neurons display increased excitability and exhibit more asynchronous discharge. The activity of a number of subcortical neurotransmitter systems from several brain regions outside the thalamus can directly affect cortical activity patterns. These neurotransmitter systems are generally targets of pharmacological intervention or participate in neurological disease states. The EEG trace comprises 4 primary rhythms: alfa (α), beta (β), theta (θ) and delta (δ), which differ in frequency and amplitude. Caffeine effect on brain asymmetry will be studied in this work. The study was realized by means of Fourier spectral frequency analysis (Fast Fourier Transformation) of the EEG signal on anesthetized rats. All 3 doses of caffeine increased the global wave power of brain activity compared to the control group. All 3 doses of caffeine reduced the number of peaks for the 0.5-4 Hz frequency band, with the intermediate dose of caffeine having such an effect in the 4-7 Hz frequency band and the high dose of caffeine for the 23-33 Hz frequency band. The group that received high doses of caffeine showed an increase of the percentage of delta waves, with a concurrent decrease of the percentage of alpha1, alpha2, beta and theta 2 compared to the control group. Low-dose caffeine produced positive values of left-right difference in brain electrical activity (left predominance) for the 0.5-5 Hz and 7.8-10.3 Hz frequency intervals. The group that received high-dose caffeine exhibited a left hemisphere dominance for the 0.5-1.5 Hz; 13.9-14.1 Hz and 19-20 Hz frequency ranges while right dominance was present in the 1.7-13.9 Hz, 15-19 Hz and 21-25 Hz frequency ranges. In conclusion, all doses of caffeine modified the global power of the brain as well as the number of peaks on the frequency range of 0.5-4 Hz. The higher dose of caffeine modified the percentage of alpha 1, alpha2, beta, delta and theta2 waves compared to the control group. The group that received 150 mg caffeine/ kg.b.w. recorded a reversal in the cerebral asymmetry of rats in the 1.7-13.9 Hz, 15-19 Hz and 21-25 Hz frequency ranges.

## Introduction

The history of recording EEG activity begins with Galvani’s experiments in 1791 with ‘‘animal electricity’’ [**1**,**2**]. Since then, important remarks have included Richard Caton’s observations of ‘‘continuous spontaneous electrical activity’’ [**3**]. Furthermore, the work of Hans Berger with human scalp recordings were essential in the evolution of EEG methods, during which the term ‘‘Elektrenkephalogramm’’ was established [**4**-**6**]. EEG recordings of electrical activity of the human brain are traditionally acquired with non-invasive electrodes placed on the scalp. In animal models, electrocorticographic (ECoG) recordings are most frequently used where electrodes are placed, on or below the dura mater, or directly within the cortex. These recordings reflect the gross electrical activity emanating from synaptic currents of individual neurons across large cortical areas. Given the similarities in brain structure, it is reasonable that measurements of cortical activity within the brain are generally translatable across species [**7**]. The EEG exhibits a spectrum of oscillation frequencies, which are modulated across the sleep-wake axis [**8**]. During periods of cortical activation, waking, and higher EEG frequencies, neurons display increased excitability and exhibit more asynchronous discharge. These patterns of spontaneous EEG activity observed throughout the circadian cycle can be classified into a number of states [**9**]. A strong proponent for the use of human qEEG in psychiatric and pharmaco-EEG research has been TM Itil and collaborators [**10**-**13**]. The activity of a number of subcortical neurotransmitter systems from several brain regions outside the thalamus can directly affect the cortical activity patterns. These neurotransmitter systems are generally targets of pharmacological intervention or participate in neurological disease states. The impact of several drugs has been shown to have similar effects on the EEG patterns of animal models and humans for a number of drug classes [**7**]. This contains an evaluation of the influence of different doses of caffeine on electrocorticograph recordings.

Electroencephalography is an electrophysiological method of exploring the central nervous system. The cortical biopotentials (spontaneous or artificial, induced by natural or artificial excitants) have varied graphical representations, in relation to the physiological or pathological state of the subject, age, neuroendocrine status, activity that is being undergone, presence, or absence of emotional tension. The EEG records the electrical activity of the superficial layers of the cerebral cortex, where neurons have less cytoplasm. The variations in potential recorded are generated by currents that are formed by the inversions of the electrical dipoles at the level of cortical pyramidal cells. These cells receive synapses from layers 1 and 4 (through dendrites or the neuronal body). The potential has a very short duration (0.5ms) and the conduction speed is high, thus the dipole is mainly formed by modifications in potential at synaptic level, which have a longer duration (6ms). The electrical activity recorded by the EEG has its origin in the metabolic and functional activity of the cortical neurons, and also in the complex subcortical neuronal networks, which influence the activity of the cerebral cortex, such as the reticulate substance and the thalamus, by means of feedback. The EEG trace comprises 4 primary rhythms: alfa (α), beta (β), theta (θ) and delta (δ), which differ in frequency and amplitude. The rhythm is made up of waves of the same type that are emited with a constant frequency, and is defined by these important parameters: frequency, amplitude, morphology, topographical localization. For a change in potential in the interior of the brain to be recorded on the EEG it has to be the result of a neuronal activity more or less sincronous that induces changes in activity in an area of at least 6 cm2 of the cortex surface. In the relaxed adult, with the eyes closed, the EEG model mainly showed an alfa rhythm, with sincronous symetric waves, with a duration of 0.5 to 3 s, a frequency of 8-12 Hz, and an amplitude of 50 μV, more pronounced in the nondominant hemisphere. The beta rhythm has a frequency of 12-30 Hz (beta 1: 12-18 Hz and beta 2: 18-30 Hz) with a smaller amplitude of 5-30 μV, being found mainly in toddlers and in the frontal regions in adults. This rhythm is suppressed during motor activities. The theta rhythm has a frequency of 4-8 Hz, with an amplitude of 40-70 μV, and can be found in children, or in the hippocampic regions in animals. In adults, it appears on the EEG in drowsiness states and is not blocked by luminous or proprioceptive stimuli. The delta rhythm has a frequency of 1-4 Hz and a big amplitude (300 μV) and can be recorded in young children in the occipital region, replacing the alpha rhythm. In adults, it can be recorded during deep sleep. In other situations, it has a pathological significance (expansive processes in the cranium, infections, or coma). Apart from these four main rhythms, there are numerous others that have been described, which are differentiated by location, morphology, physiopathological significance and are included in the so-called secondary rhythms. EEG traces can vary with age, hormonal variations, or emotional states. Desynchronization can be produced by numerous factors such as decrease of the core body temperature, blood glucose concentration, increase of the CO2 partial pressure in the arterial blood, various stimuli (visual, sensory, concentration), lesions of the thalamic nuclei, or lesions of other structures possibly implicated in the synchronization process. The EEG exam can be used to confirm the clinical diagnosis of epilepsy and can help classify it, and also can help show other modifications that could indicate structural lesions of the brain. To maximize the diagnostic value of the EEG, the exploration must be done with different assemblies, it has to have a minimum initial duration of 5 minutes at rest, has to be recorded multiple times, the examiner needing to be a specialist with a high degree of experience.

The activation methods of electrical activity of the brain are numerous and are based on the principle of modifying the brain’s reactivity, decreasing its excitability threshold, stimulating the neuronal excitability, or inserting a reflex stimulus. The activation methods can be: drug induced (pharmacological), or physiological (hyperventilation, luminous intermittent stimulation, physiological sleep stimulation or deprivation of sleep). 

Initially, it was considered that both cerebral hemispheres have similar functions, as do other pair organs in the body. Later on, the association of aphasia with the left hemisphere produced the assumption that comparable lesions of the cerebral hemispheres produce similar effects, with the exception of some neuropsychiatric effects. There are also facts that prove that some motor and sensory asymmetry exists between the two sides of the brain. The first notion of a possible specialization of one of the two cerebral hemispheres for a superior mental function has been formulated by Marc Dax in 1836, but the notion of cerebral dominance was established later on. The hypothesis regarding cerebral biochemical asymmetry can be sustained by the experiments undergone by Klementev BJ and Vartanian GA [**14**]. A unilateral cerebral lesion can lead to the appearance of an oligopeptide factor of complete asymmetry (FCA) which, when injected intracisternally, has a lateralized effect at spinal cord level. Two such factors have been identified, for each hemisphere. Cerebral metabolic asymmetry can be linked to the phenomenon of diaschisis. The term includes neuronal metabolism and cerebral blood flow depression caused by a dysfunction of an anatomically separated region (e.g.: in the opposite hemisphere) but functionally linked to the injured area (Brunberg). 

The study of interhemispheric asymmetry started from clinical observations that suggested a functional difference between the two hemispheres. The aspects initially observed concerning language were afterwards proven true also for visual, auditory, sensory perception as well as the motor function. The integration of the complex processes that represents language is undergone in the dominant hemisphere (frequently the left one). Speaking disorders are an important sign for localizing a stroke in the sylvian territory on the dominant hemisphere. The dominance of the left hemisphere for language exists in 80% of right-handed individuals and 50% of left-handed ones. Some lesions of the left hemisphere also determine visual impairments: difficulties in recognizing objects, colors, and graphical symbols, while the visual capacity is intact. It has been proven that in normal individuals, in the right visual field, the perception of written words is superior to that of the left side (Broca areas 19 and 39). Regarding the auditory senses, a lesion that affects the right hemisphere leads to difficulties in recognizing noise and musical patterns. Using the “Seashore” musical aptitude test on comitial patients who underwent the ablation of the left or right temporal lobe, B. Milner (1962) observed difficulties in memorizing melodies in patients who had a right lobectomy [**15**]. Vignolo (1969) showed that right lesions impair the discrimination of complex sound patterns, and left lesions determine a deficit in identifying the significance of sounds [**16**]. In normal subjects, a superiority of the left ear to identify music, sounds and noise has been observed. De Renzi formulated the hypothesis that the right hemisphere has a major role in organizing and discriminating sensory data obtained from the medium, while the left side has the role of naming and associating objects with their significance. The right side was also proven to have a role in perceiving the emotional stimuli that have their origin in words. In 1974, Babinski demonstrated that patients with lesions of the right hemisphere developed a motor deficit on the left side of the body. This information led to the idea of the existence of a region in the right hemisphere with a major role in integrating the sensory information from one side of the body. These lesions also affect the visual perception and other sensory perceptions. 

**Cerebral asymmetry in rats**

Highlighting the cerebral asymmetry found in rats can be done through EEG recordings. The asymmetry appears as a difference in electrical activity between the two-anteroposterior derivations: left and right. Quantitatively, this asymmetry can be measured by the difference in the power of the cerebral waves on different frequency bands. Rats present differences between sexes in regards to cerebral asymmetry, the right cortex being thicker than the left one in males, in females being the opposite [**17**]. It seems that this pattern is induced by the presence of gonadic hormones. Tail posture is also different, being slightly deviated to the left in males and to the right in females.

As written above, cerebral asymmetry is a known reality in both humans and animals. Caffeine effect on brain asymmetry will be studied in this work. The study was realized by means of Fourier spectral frequency analysis (Fast Fourier Transformation) of the EEG signal on anesthetized rats. By studying the influence of a certain substance on the electrical activity of the brain compared to a control group, conclusions can be formulated in regards to the mode in which this substance exerts its effect as well as the intimate mechanisms that determine cerebral asymmetry. Caffeine causes most of its biological effects by antagonizing all types of adenosine receptors (ARs). When acting as an AR antagonist, caffeine does the opposite of the activation of adenosine receptors, due to removal of the adenosinergic tonus. Chronic or acute intake of caffeine may affect ARs in different and even opposite ways. Having similar affinity for A1 and A2A Rs, acute caffeine actions on given brain region will reflect the preponderant AR activation in that area, since most of the adenosinergic tonus is exerted through that receptor. Caffeine stimulant effects are mediated by adenosine antagonism, but the central effects of caffeine are complex and diverse: inhibition of phosphodiesterase, promotion of calcium release from intracellular stores, or interference with GABA-A receptors.

## Materials and method

In order to assess the electrical activity of the brain, an experiment using the surface EEG method was performed. The experiment was done from 10AM to 6PM. After the onset of general anesthesia, each animal was fixed in the stereotactic device so that the 4 electrodes were placed on the scalp at equal distances from the bregma. The electrodes were at equal distances from one another, in contact with the scalp in 4 points. Through these electrodes, signals of the brain’s electrical activity were recorded by using a Biopac MP100 connected to a computer. Through the EEG machine and the analog to digital converter (MP100), the data was recorded on the computer. The recordings were 60 seconds long, with a sampling rate of 200 recordings/ second and on all 4 bipolar derivations (4 channels) as it follows: anterior (between the anterior left and anterior right electrodes), posterior (between the posterior left and posterior right electrodes), left (between the left anterior and left posterior electrodes) and right (between the right anterior and right posterior electrodes). The statistic processing of the EEG traces was done by using the BIOPAC Acknowledge software, by first applying an IIR low pass filter, with a superior limit at 33.66 Hz, and afterwards by applying the Fourier transformation. 

**Animals used**

The experiment was done on 4 groups of 6 Wistar male rats each, with a weight of 250-300g. The animals were provided by the University’s biobase. The groups of rats were brought to the laboratory 24 hours before starting the test and were maintained in standard environmental conditions with *ad libitum* access to food and water. The animals were housed in Plexiglas cages (with sawdust litter), each cage containing 4 animals. Ambient temperature was between 21 and 24 °C and relative humidity was maintained between 45-60%. All experiments were performed in accordance with European Directive 86/609/EEC/24.11.1986 and the Romanian Government Ordinance 37/ 30.01.2002 regarding the protection of animals used for experimental and other scientific purposes. The test was carried out after the approval of the Ethics Commission of the University was obtained. 

The **substances used** were the following:

- Caffeine in doses 1.5 mg/ kg.b.w., 30 mg/ kg.b.w. and 150 mg/ kg.b.w. administrated 1 hour before EEG recordings

- Urethane in dose 1g/ kg.b.w. administrated 2 hours before EEG recordings

- Saline solution in dose 0.1 ml/ 100 g.b.w.

The doses of caffeine did not exceeded 1/ 10 of the LD50. All substances were injected intraperitoneally. Sigma Aldrich provided all substances.

The concentration of the caffeine solution was calculated in order to administer a volume of 0.1 ml/ 100 g rat.

**Parameters measured**

The resulting data, which expressed the power of the cerebral waves, had a spectrum of 0-100 Hz. After removing the values that were not between 0.5 and 35 Hz, we introduced the data into Microsoft Excel, and calculated the relative power as a ratio between the power corresponding to each frequency over the total power on that derivation. Mean values were calculated for each test group. Cerebral asymmetry was demonstrated by calculating the difference between the relative powers obtained on the left channel and the right channel, and calculating the mean values for each test group. An even better clarity of the graphs was obtained by using trend lines of the relative powers on 0.25 Hz intervals on the 0.5-34 Hz interval, or 0.06 Hz on each frequency domain (delta, theta, etc.).

**Statistical analysis**

The statistic processing of the EEG traces was done by using the BIOPAC Acknowledge software, firstly by applying an IIR low pass filter, with a superior limit at 33.66 Hz and secondly by applying the Fourier transformation. 

## Results and discussions

**Fig. 1:**
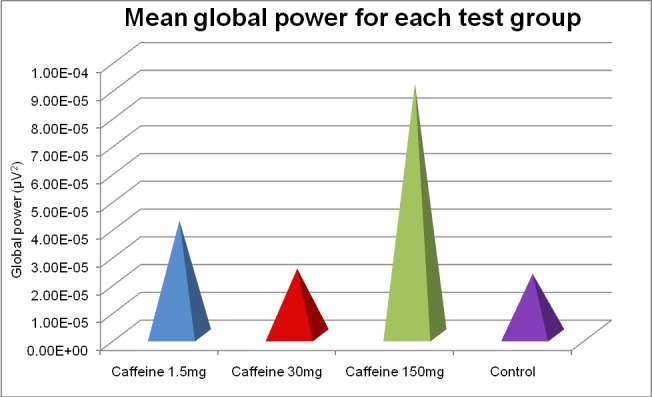
Mean global power for each test group

**Fig. 2:**
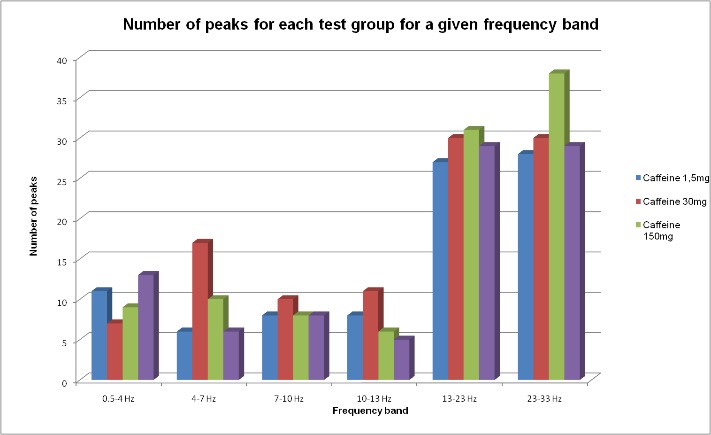
Number of peaks for each test group for a given frequency band

As seen in (**Fig. 1**,**2**) all 3 doses of caffeine increased the global wave power of brain activity compared to the control group. Even though the results were not statistically significant regarding the intermediate dose of caffeine compared to the control group (2.82 vs. 2.75), the high dose and the low dose of caffeine recorded a statistically significant increase in global wave power compared with the control group (3.87 and 8.5 vs. 2.75).

In the frequency range of 0.5-4 Hz, all 3 doses of caffeine decreased the number of peaks. The decrease was statistically significant for an intermediate dose of caffeine compared to the control group. In the frequency range of 4-7 Hz, the group which received 30 mg caffeine/ kg.b.w. presented a significant increase of the number of peaks compared to the control. In the 7-10 Hz band, none of the 3 groups receiving different doses of caffeine did not exhibit a significant change in the number of peaks compared to the control. On the frequency range of 23-33 Hz, only the group that received caffeine in a dose of 150mg/ kg.b.w showed a statistically significant increase of peaks compared to the control group.

As shown in **Table 1**, the group that received 150 mg caffeine/ kg.b.w. presented an increase in the percentage of delta waves compared to the control group (58.42 vs. 23.87). The delta rhythm is the type of brain activity with a very low frequency (4 Hz) and high amplitude, which frequently appears in infants during wakefulness. In adults, it is usually recorded frontally, while in children and infants, it is mainly present in the occipital region. This rhythm occurs physiologically during non-REM sleep, specifically during slow-wave sleep (stages 3 and 4 NREM sleep). Although initially thought to represent a marked decrease of brain activity, recent studies have shown that in reality, during NREM sleep, an increased level of brain activity is present, possibly associated with the transfer from short term to long-term memory of information gained throughout the day. Psychiatric disorders such as schizophrenia, produce changes in phases 3 and 4 NREM sleep. Specifically, there appears to be a strong association between reduced delta activity during sleep and the existence of negative symptoms of schizophrenia.

The percentage of theta2 waves was significantly decreased in the case of animals that received high-dose caffeine compared to the control group (4.23 vs. 8.29). With a frequency between 4 and 7 Hz, theta rhythm is slow, associated with inactivity of certain cortical regions. It is considered physiological in young children. It can be present in adults only in particular situations, such as sleep or waking states, meditative and/ or contemplative states. Interestingly, these rhythms occur when a person is trying to refrain from certain actions [**18**]. In rats, theta rhythm is considered a hippocampic rhythm with a frequency between 6 and 10 Hz which occurs when the animal is engaged in motor or olfactory exploration or during REM sleep. When the rat stands still in a state of wakefulness, these theta waves are much lower in power than normal.

The rats injected with 150 mg caffeine/ kg.b.w. showed a decrease in the percentage of alpha1, alpha2 and beta waves compared to the control group.

**Table 1:** Rats injected with 150 mg caffeine/ kg.b.w.

	Delta (0.5-3.9Hz)	Theta-1 (4.0-5.9Hz)	Theta-2 (6.0-7.9Hz)	Alpha-1 (8.0-9.9Hz)	Alpha-2 (10-12.9Hz)	Beta (13-25Hz)	Total
Caffeine 1.5mg	35,32	10,60	8,35	7,72	9,74	28,27	100
Caffeine 30mg	26,75	10,22	7,86	7,43	10,83	36,91	100
Caffeine 150mg	58,42	8,35	4,23	3,36	5,60	20,04	100
Control	23,87	9,58	8,29	8,12	11,25	38,90	100

The alpha rhythm, called “posterior basal rate” or “posterior dominant rhythm”, mainly occurs in the occipital region of both cerebral hemispheres, more pronounced in the dominant hemisphere. The alpha rhythm occurs when the subject’s eyes are closed and he is in a state of cognitive relaxation. Also, as it is easily predictable, alpha rhythm disappears with physical or intellectual activity. The beta rhythm is identified in frontal regions, where it is associated with a motor-like behavior, the desire to initiate movement. This rhythm decreases in amplitude simultaneously with the active movements. Brain activity with beta waves, characterized by a high frequency, a small amplitude, and quasi-irregularity, is suggestive for an intensive cognitive activity or for anxious moods, while the same rate dominated by beta waves, but this time in a regular pattern, with high amplitudes, is an adverse reaction of benzodiazepines observed on EEG recordings.

For the analysis of inter-hemispheric brain asymmetry relative power differences between the left and right hemispheres were initially calculated by using “moving averages” on intervals of 0.25 Hz. Positive values of this parameter would denote a left dominant hemisphere for a given frequency band, whilst negative values would be present in right dominant hemisphere subjects.

**Fig. 3:**
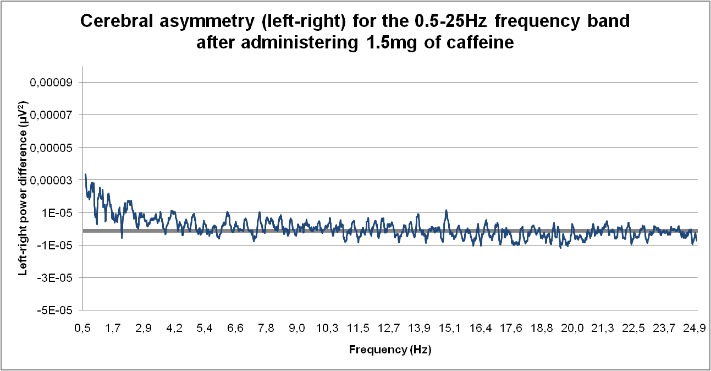
Cerebral asymmetry (left-right) for the 0.5-25 Hz frequency band after administering 1.5mg of caffeine

In the group that received low-dose caffeine, predominantly positive values were observed (left predominance) characteristic of left-right asymmetry for the 0.5-5 Hz and 7.8-10.3 Hz frequency intervals. Most significant negative values of the left-right differences of relative power (right hemisphere dominance) were recorded in the range from 15 to 17.6 Hz and 18.8 to 21.3 Hz for the group that received 1.5 mg caffeine/ kg.b.w.. As it can be seen in the accompanying charts, compared to the control group, low-dose caffeine canceled brain electrical asymmetry in the range 16-20 Hz and 22-25 Hz.

In rats which received 30 mg caffeine/ kg.b.w., positive values were recorded for cerebral asymmetry for frequency ranges from 0.5 to 1.7 Hz; 3 to 4.2 Hz and 7.8 to 9 Hz, while negative values were obtained in the 5-6 Hz, 13.9-15 Hz; 17.6-18.8 Hz and 20-24.9 Hz frequency bands.

**Fig. 4:**
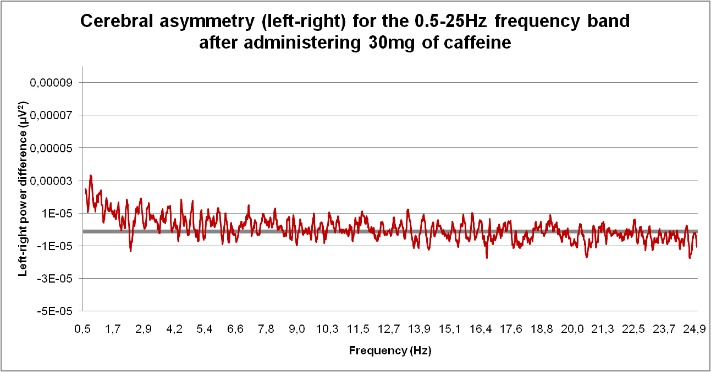
Cerebral asymmetry (left-right) for the 0.5-25 Hz frequency band after administering 30mg of caffeine

The group that received 150 mg caffeine/ kg.b.w. recorded positive values of cerebral asymmetry in the 0.5-1.5 Hz; 13.9-14.1 Hz and 19-20 Hz frequency ranges. Negative values were recorded in the 1.7-13.9 Hz, 15-19 Hz and 21-25 Hz frequency ranges. The medium dose of caffeine canceled brain electrical asymmetry in the 4-6 Hz band, while high dose caffeine canceled brain electrical asymmetry in the 4-6 Hz; 9-10, 10-12 and 16-19 Hz ranges, compared to the control group.

**Fig. 5:**
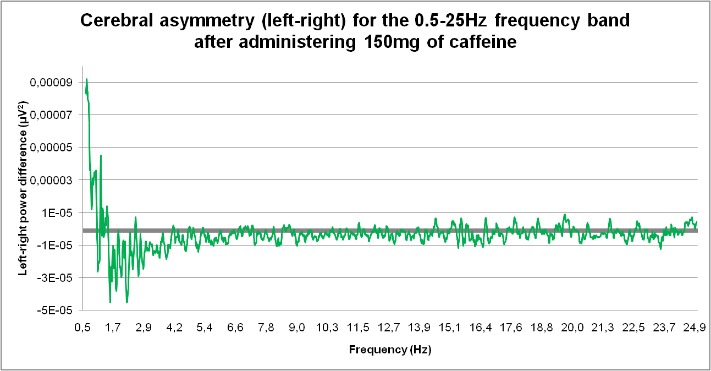
Cerebral asymmetry (left-right) for the 0.5-25 Hz frequency band after administering 150mg of caffeine

The control group reported a left-right brain asymmetry in the 1.7-1.8 Hz; 2.9-4.1 Hz, 4.3 to 6 Hz, 9-10.3; 10.3-12.7; 16.4-20.5 and 22.5-25 Hz ranges. Negative values showing right side dominance were recorded for the 1.3-1.6 Hz, 4.2-4.5 Hz; 6-6.6 Hz, 7.8-9 Hz, 11-11.5 Hz, 16.4-7.7 Hz and 23.7-24 Hz intervals.

**Fig. 6:**
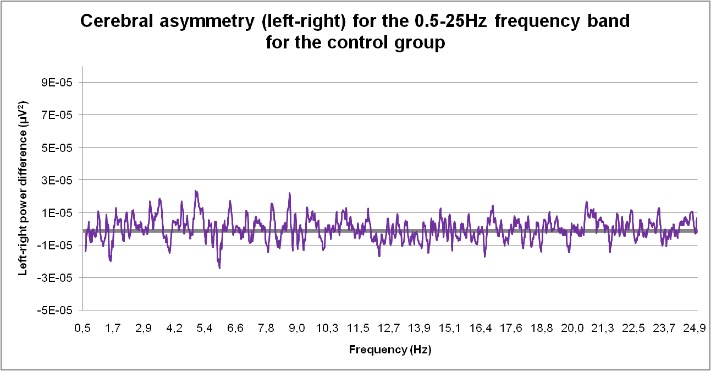
Cerebral asymmetry (left-right) for the 0.5-25 Hz frequency band for the control group

## Conclusions

1. All 3 doses of caffeine increased the global wave power of brain activity compared to the control group.

2. In the frequency range of 0.5-4 Hz, all 3 doses of caffeine decreased the number of peaks.

3. In the frequency range of 4-7 Hz, the group that received 30 mg caffeine/ kg.b.w. presented a significant increase in the number of peaks compared to the control. In the 7-10 Hz band, none of the 3 groups receiving different doses of caffeine did exhibit a significant change in the number of peaks compared to the control.

4. In the frequency range of 23-33 Hz, only the group that received caffeine in a dose of 150mg/ kg.b.w showed a statistically significant increase in the number of peaks compared to the control group. 

5. The group that received 150 mg caffeine/ kg.b.w., presented an increase in the percentage of delta waves compared to the control group.

6. The percentage of theta2 waves was significantly decreased in the case of animals that received high-dose caffeine compared to the control group.

7. The rats injected with 150 mg caffeine/ kg.b.w., showed a decrease in the percentage of alpha1, alpha2 and beta waves compared to the control group.

8. Most significant negative values of the left-right differences of relative power (right hemisphere dominance) were recorded in the range from 15 to 17.6 Hz and 18.8 to 21.3 Hz for the group that received 1.5 mg caffeine/ kg.b.w.

9. In rats which received 30 mg caffeine/ kg.b.w., positive values were recorded for cerebral asymmetry for frequency ranges from 0.5 to 1.7 Hz; 3 to 4.2 Hz and 7.8 to 9 Hz, while negative values were obtained in the 5-6 Hz, 13.9-15 Hz; 17.6-18.8 Hz and 20-24.9 Hz frequency bands.

10. The group that received 150 mg caffeine/ kg.b.w., recorded positive values of cerebral asymmetry in the 0.5-1.5 Hz; 13.9-14.1 and 19-20 Hz frequency ranges. Negative values were recorded in the 1.7-13.9 Hz, 15-19 Hz and 21-25 Hz frequency ranges.

11. The low-dose of caffeine canceled brain electrical asymmetry in the range 16-20 Hz and 22-25 Hz.

12. The medium dose of caffeine canceled electrical brain asymmetry in the 4-6 Hz band while high dose caffeine canceled brain electrical asymmetry in the 4-6 Hz; 9-10 Hz, 10-12 Hz and 16-19 Hz ranges compared to the control group.
